# Retrospective evaluation of possible renal toxicity associated with continuous infusion of vancomycin in critically ill patients

**DOI:** 10.1186/2110-5820-1-26

**Published:** 2011-07-19

**Authors:** Herbert D Spapen, Karin Janssen van Doorn, Marc Diltoer, Walter Verbrugghe, Rita Jacobs, Nadia Dobbeleir, Patrick M Honoré, Philippe G Jorens

**Affiliations:** 1 Department of Intensive Care, University Hospital, Vrije Universiteit, Brussels, Brussels, Belgium; 2 Department of Nephrology, Antwerp University Hospital, University of Antwerp, Edegem, Belgium; 3 Department of Intensive Care, Antwerp University Hospital, University of Antwerp, Edegem, Belgium

## Abstract

**Background:**

Continuous infusion of vancomycin is increasingly preferred as an alternative to intermittent administration in critically ill patients. Intermittent vancomycin treatment is associated with an increased occurrence of nephrotoxicity. This study was designed to determine the incidence and risk factors of acute kidney injury (AKI) during continuous infusion of vancomycin.

**Methods:**

This was a retrospective, observational, two-center, cohort study in patients with microbiologically documented Gram-positive pneumonia and/or bacteremia and normal baseline renal function. Vancomycin dose was adjusted daily aiming at plateau concentrations of 15-25 μg/mL. AKI was defined as an increase in serum creatinine of 0.3 mg/dL or a 1.5 to 2 times increase from baseline on at least 2 consecutive days after the initiation of vancomycin. Primary data analysis compared patients with AKI with patients who did not develop AKI. A binary logistic regression analysis using the forward stepwise method was used to assess the risk factors associated with AKI.

**Results:**

A total of 129 patients were studied of whom 38 (29.5%) developed AKI. Patients with AKI had higher body weight (77.3 ± 15 vs. 70.5 ± 15.2 kg; *p *= 0.02), more diabetes (79% vs. 54%; *p *= 0.01), and a higher vasopressor need (87% vs. 59%; *p *= 0.002). Serum vancomycin levels, body weight, and SAPS 3 score were identified as variables contributing to AKI. The incidence of AKI increased substantially when treatment duration was prolonged (14.9 ± 9.8 vs. 9.2 ± 4.9 days; *p *= 0.05) and plasma levels exceeded 30 μg/mL.

**Conclusions:**

AKI is frequently observed during continuous vancomycin infusion, particularly when conditions that cause acute (shock) or chronic (diabetes) renal dysfunction are present and vancomycin levels above target range are achieved. Although this study challenges the concept that continuous vancomycin infusion might alleviate the risk of nephrotoxicity in critically ill patients, a direct relationship between vancomycin and nephrotoxicity remains to be proven.

## Background

Several reasons might explain why conventional twice-daily dosing of vancomycin often fails to obtain microbiological and clinical cure in patients with *Staphylococcus aureus *(SA) pneumonia and bloodstream infections: poor penetration in infected and/or ventilated lung tissue, a subtle but significant increase in minimal inhibitory concentration (MIC) over time, also referred to as the MIC "creep," and the emergence of heteroresistant strains [1,2]. These observations have prompted experts to decrease the breakpoint of vancomycin susceptibility from 4 to 2 μg/mL and to recommend targeting serum vancomycin trough levels of 15-20 μg/mL for the treatment of methicillin-resistant SA (MRSA) pneumonia [3]. However, attempts to optimize vancomycin exposure and hence antibacterial effectiveness by using higher loading and maintenance doses are associated with an increased incidence of nephrotoxicity [4].

Continuous infusion of vancomycin has been proposed as a logistically and pharmacodynamically more convenient alternative to intermittent administration [5]. However, the risk of developing acute kidney injury (AKI) during continuous vancomycin infusion remains poorly examined, especially in the critically ill. Vancomycin pharmacodynamics in this population is challenged by large variations in distribution volume during resuscitation, enlargement of the extracellular space, and significant fluctuations in renal clearance [6]. Intensive care unit (ICU) patients also are exposed to a wide array of potential nephrotoxic agents, which increases the risk for vancomycin-associated nephrotoxicity.

Defining the incidence and risk factors of AKI associated with continuous vancomycin infusion is important given the availability of alternative anti-Gram-positive agents that are believed to be less nephrotoxic. We therefore studied the relationship between vancomycin steady state plateau concentrations during continuous infusion and occurrence of AKI in ICU patients with Gram-positive bacteremia and/or pneumonia. Additionally, potential risk factors for nephrotoxicity during vancomycin infusion were identified.

## Methods

A retrospective observational cohort study was conducted in the ICUs of two Belgian tertiary care hospitals: University Hospital, Vrije Universiteit Brussels; and Antwerp University Hospital, University of Antwerp. Patients who were hospitalized from January 1, 2008 until November 31, 2009 were included in the study if they were older than aged 18 years, had an absolute neutrophil count ≥ 1,000 cells/mm^3^, had a microbiologically documented Gram-positive pneumonia and/or bacteremia, received a continuous infusion of vancomycin for at least 5 days, and had a baseline serum creatinine < 1.5 mg/dL. Patients were excluded if diagnosed with cystic fibrosis, bronchiectasis, meningitis, or polymicrobial infection, if intravenous contrast dye was given within 7 days of the start of vancomycin treatment, and if data with regard to vancomycin and creatinine serum levels were missing. The study was approved by both Hospitals' Institutional Review Boards. In view of the retrospective and observational nature of the study with no interventions performed, the need for informed consent was waived.

A laboratory computed database was searched to identify all patients with community-, hospital-, or healthcare-acquired pneumonia and bacteremia that had been treated with continuous vancomycin infusion. For all patients, the following information was retrieved from their medical records: age, gender, weight, serial serum creatinine levels, simplified acute physiology score (SAPS) 3, and daily vancomycin plateau serum concentrations. Additionally, data were collected on concomitant exposure to potential nephrotoxic drugs (angiotensin converting enzyme inhibitors (ACEi), angiotensin II receptor antagonists (AT-IIra), nonsteroidal anti-inflammatory drugs (NSAIDs), aminoglycosides and immunosuppressants (calcineurin inhibitors (ciclosporin, tacrolimus) and sirolimus), and underlying (diabetes) or acute (shock) risk factors for AKI.

Nephrotoxicity was defined by using the Acute Kidney Injury Network (AKIN) classification [7] as an increase in serum creatinine level of 0.3 mg/dL or a 1.5 to 2 times increase from baseline, whichever was greater, (i.e., AKIN stage 1) on at least 2 consecutive days during the period from initiation of vancomycin to 72 h after completion of therapy. Urine output was not measured as part of AKIN staging due to concerns about its dependency on uncontrolled "extrarenal" events, such as volume status, treatment with loop diuretics and vasoactive agents, and release of antidiuretic hormone. Nephrotoxic drug exposure was considered to be relevant only when present during vancomycin infusion and before increase in serum creatinine. Shock was defined as the need for vasopressor therapy to obtain a mean arterial pressure ≥70 mmHg in a patient who was adequately fluid-resuscitated with crystalloid and colloid solutions.

All patients received vancomycin as a 15-mg/kg loading dose infused over 60 min, immediately followed by a continuous infusion of 30 mg/kg/day. Treatment was adjusted afterwards based on plateau serum vancomycin concentrations by increasing or decreasing the speed of the volumetric infusion device so that the daily dose was increased or decreased by 500 mg [5]. Treatment was designed to obtain a plateau vancomycin level between 15 and 25 μg/mL. When vancomycin concentrations exceeded 30 μg/mL, vancomycin infusion was interrupted for 6 h, after which plasma concentrations were reevaluated. This procedure was repeated until vancomycin levels returned within target range. Vancomycin and creatinine levels were measured daily at 8:00 A.M. in all patients. Vancomycin concentrations were assessed by quantitative enzyme immunoassay using the Vitros 5.1 FS Chemistry System (Ortho Clinical Diagnostics, Inc., Beerse, Belgium) or the Dimension Vista (Siemens Healthcare Diagnostics GmbH, Eschborn, Germany) and classified in three groups: < 25 μg/mL, 25-30 μg/mL, and > 30 μg/mL.

### Statistical analysis

Statistical measurements were performed by using SPSS software, version 19.0 for Windows (SPSS, Inc., Chicago, IL). Primary data analysis compared patients with AKI with patients who did not develop AKI. Continuous variables were assessed with the Student's *t *test for normally distributed variables and the Mann-Whitney *U *test for nonnormally distributed variables. The chi-square test was used to compare categorical variables. Values were expressed as means ± standard deviations (continuous variables) or as a percentage of the group from which they were derived (categorical variables). *P *values were two-tailed and considered to indicate statistical significance at the < 0.05 level.

A binary logistic regression analysis using the forward stepwise method with occurrence of AKI as dependent variable was performed, including the following parameters: presence of diabetes or shock, use of aminoglycosides, ACEi or AT-IIra, NSAIDs, and immunosuppressants. All potentially explanatory variables in this regression were tested on multicollinearity using the collinearity diagnostics by linear regression calculating the variance inflation factor.

## Results

**Figure 1:**
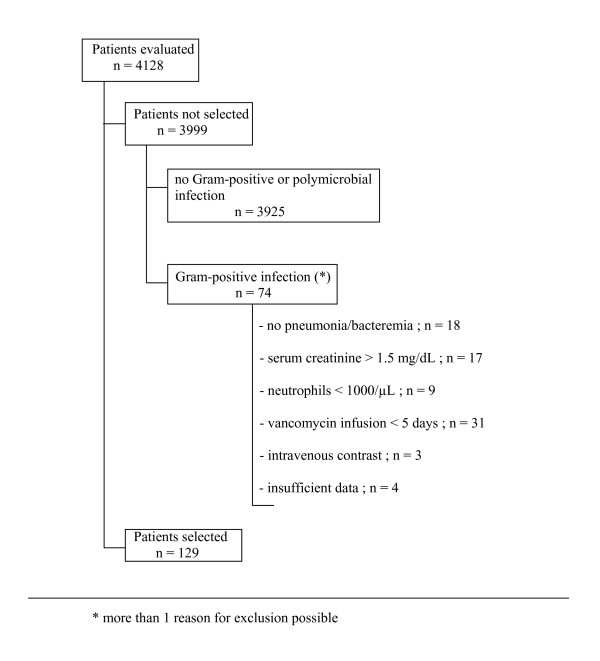
**Patient selection**.

During the study period, a total of 4,128 patients were hospitalized in the ICUs of both hospitals. Of these, 129 patients with Gram-positive infection (MRSA (n = 82; 63.6%), methicillin-resistant coagulase-negative *Staphylococci *(n = 40; 31%), *Enterococcus *spp. (n = 7; 5.4%) were selected for the study (Figure 1). Patients' characteristics are depicted in Table 1. Seventy-eight patients had pneumonia, 38 had bacteremia, and 13 had both pneumonia and bacteremia. Thirty-eight subjects (29.5%) developed AKI during vancomycin treatment. No relationship was found between type of infection and incidence of AKI (data not shown). Baseline creatinine values were comparable between patients with and without AKI (0.9 ± 0.19 mg/dL vs. 0.8 ± 0.35 mg/dL; *p *= 0.12). Age, gender, and severity of illness also did not differ between patients with and without AKI. Patients who developed AKI had a higher lean body weight and were more likely to have diabetes or shock. In both groups, a comparable number of potential nephrotoxic agents were used concomitantly with vancomycin. No association was found between this nephrotoxic medication and the occurrence of AKI. Patients with AKI received vancomycin for a more prolonged time period (14.9 ± 9.8 vs. 9.2 ± 4.9 days; *p *= 0.05). Creatinine concentrations in this group increased after a mean duration of vancomycin treatment of 116 h ± 82 h (range, 53-402 h). Except for adjusting treatment at levels > 30 μg/mL, vancomycin was never discontinued or switched to an alternative agent. Mortality was higher in patients who developed AKI (53% vs. 20%; *p *= 0.01).

**Table 1:** Patient characteristics

Variable	No AKI (n = 91)	AKI (n = 38)	P value
SAPS 3	67 ± 15	68 ± 14	0.63
Age, yrs	60.8 ± 14.6	62.6 ± 15.9	0.53
Weight, kg	70.5 ± 15.2	77.3 ± 15.0	0.02*
Gender, % male	51.6	60.5	
Concomitant medication			
Aminoglycosides, n (%)	39 (43)	12 (32)	0.32
ACEi or AT-IIra, n (%)	17 (19)	7 (18)	1
NSAIDs, n (%)	4 (4)	1 (3)	1
Immunosuppressants, n (%)	2 (2)	0 (0)	1
Comorbidities			
Diabetes, n (%)	49 (54)	30 (79)	0.01*
Shock, n (%)	54 (59)	33 (87)	0.002*

AKI = acute kidney injury; SAPS = Simplified Acute Physiology Score;  ACEi = angiotensin converting enzyme inhibitors; AT-IIra = angiotensin-II receptor antagonists; NSAIDs = non steroidal anti-inflammatory drugs

* P level < 0,05 is considered significant

The logistic regression multivariate analysis identified vancomycin plasma level (*p *< 0.001), weight (*p *= 0.002), and SAPS 3 (*p *= 0.024) as independent variables associated with AKI. The probability of AKI can be calculated as *P *= 1/1+ e^-logit ^with logit = -6.54 + 0.055 × SAPS 3 + 0.067 × weight (kg) - 5.888 × 1 (if vancomycin level < 25 μg/mL) - 3.178 × 1 (if vancomycin level < 30 μg/mL). A higher logit value results in an increased probability of AKI. The highest logit is associated with vancomycin levels exceeding 30 μg/mL and is further increased when SAPS 3 values or body weight rise. However, the three variables differ in relative weight. Indeed, a 10 kg or 1 point increase in respectively lean body weight or SAPS 3 will result in a 0.55 and 0.065 logit increase. In contrast, a vancomycin level between 25 and 30 μg/mL causes a logit increase of 3.1718. Changes in the accuracy of the predictive model also demonstrate the relative importance of the predicting parameters. When the prediction model is based solely on the vancomycin level, the addition of SAPS 3 as a marker of disease severity and lean body weight causes only a very modest increase of the overall percentage (from 86% to 86.8%) correctly predicted AKI. Including SAPS 3 in the model containing vancomycin level and lean body weight makes the Nagelkerke R^2 ^increase from 0.637 to only 0.671. Adding any of the other observed variables to the equation did not enhance predictive power. Testing for potentially explanatory variables on multicollinearity revealed a variance inflation factor between 1.097 and 1.56, thus far below 2.5, which is considered to be the threshold value for concern of collinearity. The relationship between the diabetes state and variables, such as age, body weight, and SAPS 3, was closely examined, because it seems reasonable to assume that diabetics are older, are more overweight, and are likely to be more severely ill. However, no significant correlation was identified. The seeming paradox that diabetes and shock are found to be associated with AKI in univariate analysis whilst multivariate analysis demonstrated no contribution of these parameters to the accuracy of the model is due to differences in methodology.

**Figure 2:**
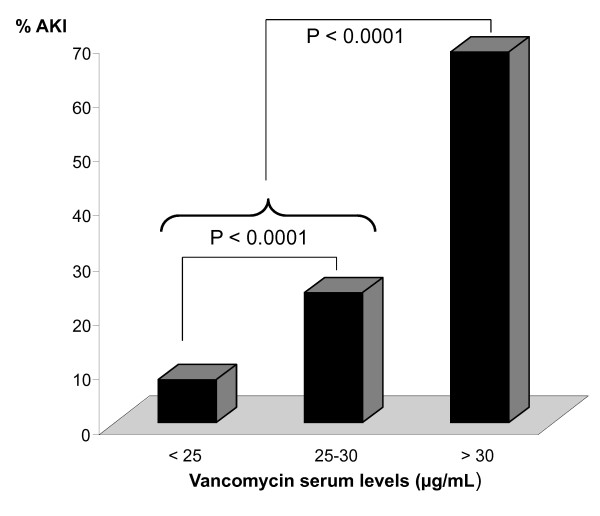
**Vancomycin serum levels in patients who develop acute kidney injury (AKI) during vancomycin infusion**.

The distribution of vancomycin concentrations is shown in Table 2. Within the group who developed AKI, a distinct association was found between vancomycin levels and the occurrence of AKI (Figure 2). AKI was more frequently found in patients with vancomycin levels between 25-30 μg/mL than in those with levels not exceeding 25 μg/mL (9 (24%) vs. 3 (8%) patients; odds ratio 9.75, confidence interval 2.41-39.52; *p *< 0.0001). The incidence of AKI sharply increased when vancomycin attained concentrations > 30 μg/mL compared with patients whose values remained below this level (12 (32%) vs. 26 (68%) patients; odds ratio 30.69, confidence interval 10.49-89.83; *p *< 0.0001). No patient developed overt renal failure or needed dialysis. Serum creatinine values at discharge had returned to baseline levels in 12 of the 18 (66%) surviving patients with vancomycin-associated AKI.

**Table 2:** Vancomycin levels in patients with and without nephrotoxicity

Vancomycin concentration* (μg/mL)	No AKI (n = 91)	AKI (n = 38)
< 25	65	3
25-30	20	9
> 30	6	26

AKI = acute kidney injury,

* defined groups represent at least 2 consecutive measures within the given concentration range

## Discussion

Vancomycin is the first-line antibiotic treatment for infections caused by MRSA and often is used for treatment of other Gram-positive infections. Key factors that determine appropriate dosing of vancomycin in MRSA and staphylococcal bacteremia and pneumonia include the MIC of the infecting organism and the risk of nephrotoxicity. Pharmacological studies have determined that the parameter best predicting vancomycin activity is the 24-h area under the concentration curve over the MIC (AUC/MIC or AUIC). It is recommended that this ratio be kept between 350 and 400 to achieve a good clinical and microbiological response against MRSAs with MIC values ≤1 μg/mL [8]. To determine vancomycin effectiveness in the clinical setting, serum trough levels are measured as a surrogate of AUIC. Aiming at high trough concentrations will increase the likelihood of achieving a more optimal AUIC but also the risk to develop nephrotoxicity.

Several studies have evaluated the incidence of nephrotoxicity--defined as a more than 50% increase of serum creatinine from baseline value or an absolute increase of serum creatinine of 0.5 mg/dL--during intermittent vancomycin treatment. Two retrospective studies found that when trough levels exceeded 10 μg/mL [9] or 15 μg/mL [10], the incidence of nephrotoxicity increased by respectively 17% and 27%. In a prospective cohort study that included 80% patients with pneumonia or bacteremia, Hidayat and coworkers observed 11.6% nephrotoxicity when vancomycin dose was adjusted to achieve trough values of four to five times the MIC of the infecting MRSA strain [11]. These studies identified high vancomycin trough levels, prolonged duration of vancomycin therapy and ICU stay, and concomitant treatment with nephrotoxic agents, in particular aminoglycosides, as independent risk factors for nephrotoxicity. Our experience with continuous vancomycin infusion corroborates these findings. Moreover, the risk to develop AKI in a population consisting exclusively of critically ill ICU patients was largely determined by conditions that significantly compromise the kidney, such as diabetes and shock. Interestingly, we were unable to detect an association between vancomycin-associated AKI and the use of concomitant potentially nephrotoxic drugs. This could be explained by standard "precautions" shared by both ICUs, including short-term use of aminoglycosides under daily monitoring of trough levels and a restrictive prescription policy of NSAIDs and drugs acting on the renin-angiotensin pathway.

Continuous infusion of vancomycin has been proposed as an elegant approach for maximizing efficacy of the drug whilst avoiding development of resistance and adverse renal effects. In clinical practice, a steady state plateau plasma vancomycin concentration of 20-25 μg/mL is targeted. This concentration is selected according to available data on the drug's tissue diffusion and protein binding [12] and anticipates an eventual surreptitious MIC creep [13]. Vancomycin levels are kept within target range by therapeutic monitoring and dose adjustment according to renal function. Using this approach, however, vancomycin levels were found to be higher than 30 μg/mL in 24.8% (32/129) of our study patients. A similar observation was made by French investigators who reported trough vancomycin serum levels that exceeded 30 μg/mL in 200 of 957 (20.9%) patients who received continuous infusion [14]. From the study by Wysocki and coworkers [5], it is apparent that a substantial number of patients had steady state vancomycin plasma levels > 25 μg/mL throughout continuous treatment. These findings underscore that continuous infusion aiming at plateau levels between 20 and 25 μg/mL may expose a significant proportion of patients to higher than expected vancomycin concentrations, and hence nephrotoxicity. Ingram and coworkers indeed detected a striking difference in incidence of nephrotoxicity at a vancomycin cutoff serum level of 28 μg/mL during continuous infusion [15]. Above this level, 5 of 7 patients (71.4%) experienced a significant increase in serum creatinine levels, whereas this occurred in only 11 of 91 patients (11.6%) whose vancomycin levels remained below 28 μg/mL. This is in line with our observations that indicated a substantial increase in AKI at vancomycin levels between 25 and 30 μg/mL, which became alarming when levels exceeded 30 μg/mL.

Although we cannot exclude that higher vancomycin serum levels might reflect simple accumulation of the drug, we believe that aiming at concentrations up to 25 μg/mL with continuous infusion is harming patients. It may indicate that the actual algorithms to adapt vancomycin dosing during continuous infusion should be revised. Surprisingly, data on vancomycin dosing based on solid pharmacodynamic studies in ICU patients are scarce, in particular when renal function is decreasing. Moreover, the proposed models present important drawbacks. Information about infection type, severity of illness, resuscitation state, comorbid disease, and eventual concomitant use of nephrotoxins is not provided [16,17]. Also, creatinine clearance is assessed by means of the Cockroft-Gault formula, although it has been shown that such estimation may poorly predict vancomycin clearance in an ICU population [18]. Measurement of creatinine clearance or renal biomarkers (e.g., cystatin) may be more practical and correct alternatives to determine vancomycin dose requirements [18] but await prospective validation.

Studies that compare continuous with intermittent administration of vancomycin did not show a convincing difference in occurrence of nephrotoxicity. Ingram and coworkers retrospectively studied 167 patients with bone, joint, and skin MRSA infection on an outpatient parenteral therapy with vancomycin [19]; 12 subjects were treated with continuous infusion and 55 received intermittent vancomycin in an nonrandomized setting. Overall incidence of nephrotoxicity was 15.6%. In 40 matched pairs of patients, the incidence of nephrotoxicity when receiving continuous or intermittent treatment was 10% versus 25% (*p *= 0.14). Continuous infusion was associated with a slower onset of nephrotoxicity. Vuagnat and coworkers compared continuous with intermittent administration in respectively 23 and 21 patients with osteomyelitis, aiming at trough or plateau levels between 20 and 25 μg/mL [20]. Four (19%) patients undergoing intermittent treatment but no patients undergoing continuous treatment developed nephrotoxicity. The largest prospective study on continuous vancomycin treatment to date included 119 patients mostly suffering from pneumonia or bacteremia. Sixty-one patients were randomized to receive continuous infusion, aiming at plateau levels between 20 and 25 μg/mL [5]. The remaining 58 subjects received vancomycin intermittently to obtain trough levels between 10 and 15 μg/mL. Overall, a 20% incidence of nephrotoxicity was observed without difference between continuous and intermittent treatment (16.4% vs. 19%; *p *= 0.64). Hutschala and coworkers retrospectively evaluated 149 patients who underwent cardiac surgery [21]. Depending on ICU physicians' preference, 119 patients were treated with continuous infusion (target plateau 20-25 μg/mL) and 30 patients received intermittent treatment (target trough 15 μg/mL). Approximately 35% of patients in each group had pneumonia or bacteremia. Nephrotoxicity was defined according to the AKIN criteria. This study found the highest incidence of nephrotoxicity (29.5%) but again no difference between continuous and intermittent administration (27.7% vs. 36.7%; *p *= 0.3). Interestingly, we observed nephrotoxicity in only 8% of patients whose vancomycin levels remained between 15 and 25 μg/mL. This is considerably lower than the 19% to 35% incidence of nephrotoxicity reported at similar trough levels during intermittent vancomycin administration [5,10,20,21].

It must be emphasized that attaining AKIN stage I, by definition, does not signify renal failure. In fact, none of our patients who developed vancomycin-associated renal toxicity required renal replacement therapy (RRT) during their ICU stay, nor was vancomycin discontinued or replaced by alternative agents. Jeffres and coworkers also reported no need for RRT in patients with severe pneumonia who developed nephrotoxicity during intermittent vancomycin treatment [10]. On the other hand, Hutschala and coworkers reported the need for RRT in 25% of patients receiving continuous or intermittent vancomycin treatment [21]. However, this study population consisted of cardiac surgery patients at high risk for developing postoperative acute renal failure with some already presenting renal dysfunction at baseline. A noticeable finding was that less patients under continuous infusion required RRT after 5 days of vancomycin treatment (23.5% vs. 30%, *p *= 0.053).

Some important limitations of our study deserve attention. We gave a vancomycin loading dose of 15 mg/kg before starting continuous infusion. Recent clinical practice guidelines of the Infectious Diseases Society of America (IDSA) recommend a loading dose of up to 25-30 mg/kg to achieve target trough concentrations rapidly in serious MRSA infections, including pneumonia and bacteremia [22]. However, clinical and safety data regarding this approach are lacking. Also, when we designed the study, the IDSA guidelines had not yet been published, and a 15-mg/kg loading dose was considered adequate by most authors [5,16,17]. In this context, it is important to note that the IDSA expert panel advises against administration of vancomycin in continuous infusion given that a clear benefit over intermittent dosing has not been demonstrated and because time above the MIC is not the primary predictor of vancomycin efficacy [22]. Although appropriate for large comparative and epidemiological studies, the AKIN classification may not be an accurate tool to assess a particular drug-related nephrotoxic effect in critically ill patients, because a rise in creatinine concentration occurs only after substantial loss of renal function. A more appropriate way to correlate vancomycin serum levels with the occurrence of kidney injury would have been to consider vancomycin concentrations in the 48 h before the onset of AKI. However, it must be conceded that any rise in creatinine levels will be delayed following "true" onset of AKI.

The retrospective nature of the study precluded the use of creatinine clearance as a more optimal measure of renal function and did not allow differentiation of infection-related or AKI-induced mortality. For the same reason, microbiological and clinical cure rates and infection-related mortality were not evaluated. This would require the identification of individual vancomycin MIC values, because these are known to influence significantly both treatment efficacy and mortality in MRSA pneumonia [23] and bacteremia [2]. Finally, it is conceivable that the higher vancomycin concentrations may have identified these patients with a hidden or progressing renal failure rather than be the cause of a subsequent increase in creatinine. Thus, the results of the present study suggest a possible vancomycin exposure-toxicity relationship but definite proof of causality will require a blinded, prospective trial.

## Conclusions

A frail balance exists between obtaining therapeutic serum levels and avoiding nephrotoxicity during continuous vancomycin infusion in critically ill patients with severe Gram-positive infections and normal baseline renal function. AKI is detected already at "conventional" serum vancomycin plateau levels but peaks alarmingly at values exceeding 30 μg/mL. AKI was not found to be associated with the concomitant use of nephrotoxic medication but is influenced rather by conditions that are known to chronically worsen (diabetes) or acutely injure (shock) renal function. Our study does not support the concept that continuous infusion with vancomycin reconciles optimization of vancomycin pharmacodynamics against SA infections with less risk of nephrotoxicity.

## Competing interests

The authors declare that they have no competing interests.

## Authors' contributions

HS and KJvD conceived the study and wrote the paper. MD provided data statistics and analysis. WV, RJ, and ND participated in data acquisition. PH participated in design, coordination, and writing. PJ participated in data interpretation, coordination, and writing.
